# Viral-driven oncogenesis in T/NK-cell lymphomas: parallels and divergences between HTLV-1 and EBV

**DOI:** 10.1007/s12185-025-04156-0

**Published:** 2026-01-07

**Authors:** Takafumi Shichijo, Jun-ichirou Yasunaga

**Affiliations:** Department of Hematology, Rheumatology and Infectious Diseases, Faculty of Life Sciences, Kumamoto University, 1-1-1, Honjo, Chuo-ku, Kumamoto, 860-8556 Japan

**Keywords:** Human T-cell leukemia virus type I, Adult T-cell leukemia-lymphoma, Epstein–Barr virus, Extranodal NK/T-cell lymphoma, Chronic active Epstein–Barr virus disease

## Abstract

Viruses induce approximately 12% of human cancers, including lymphomas. In the case of T/NK cell neoplasms, human T-cell leukemia virus type I (HTLV-1) causes adult T-cell leukemia-lymphoma (ATL), and Epstein-Barr virus (EBV) is associated with extranodal NK/T-cell lymphoma (ENKTCL) and chronic active Epstein–Barr virus disease (CAEBV). Common mechanisms for lymphoma development have been proposed. Viral genes, such as *tax* and *HTLV-1 bZIP factor (HBZ)* of HTLV-1, and *latent membrane protein 1 (LMP1)* and *BamHI A rightward transcript microRNA (miRNA-BART)* of EBV, contribute to host immune evasion and modulation of host signaling pathways, resulting in the persistence of viral-infected cells. This viral strategy is closely associated with oncogenesis. Furthermore, the long-term survival of infected cells leads to the accumulation of somatic mutations and aberrant epigenetic alterations. These events eventually lead to ATL, ENKTCL, and the lymphoma-like subset of CAEBV. Interrupting these common oncogenic mechanisms is a promising therapeutic strategy for viral-driven lymphomas with poor prognoses.

## Introduction

Infections induce 15–20% of human cancers [1]. Almost all of these cancers are viral-driven (accounting for approximately 12% of all cancers), including hepatocellular carcinoma, gastric cancer, cervical cancer, nasopharyngeal carcinoma, Kaposi sarcoma, and lymphomas [2–4]. The oncogenic viruses responsible for these cancers include hepatitis B virus, human papillomavirus, Epstein-Barr virus (EBV), Kaposi sarcoma-associated herpesvirus (KSHV)/human herpes virus 8 (HHV-8), hepatitis C virus, human T-cell leukemia virus type I (HTLV-1), and human immunodeficiency virus [2–4].

HTLV-1, EBV, and HHV-8 are well-known for inducing lymphomas and other lymphoproliferative diseases. HTLV-1 induces adult T-cell leukemia-lymphoma (ATL) [5], EBV is associated with Burkitt lymphoma, Hodgkin lymphoma (HL), post-transplant lymphoproliferative disorder, extranodal NK/T-cell lymphoma (ENKTCL), and chronic active Epstein–Barr virus disease (CAEBV) [6, 7], while HHV-8 causes Kaposi’s sarcoma, primary effusion lymphoma, multicentric Castleman’s disease, and HHV-8–positive diffuse large B-cell lymphoma (DLBCL) [7].

Many viruses share common oncogenic mechanisms. Specifically, these mechanisms involve strategies for persistent or latent infection to evade host immunity and facilitate viral replication or the proliferation of infected cells [2]. Therefore, understanding the mechanisms underlying persistent viral infection can provide insight into potential therapeutic targets for virus-associated diseases.

In this review, we summarize the recent findings on viral-driven oncogenesis in T/NK-cell lymphomas, focusing on HTLV-1 and EBV.

## Human T-cell leukemia virus type I induces adult T-cell leukemia-lymphoma

HTLV-1 was the first retrovirus discovered in humans, and the first retrovirus proven to be oncogenic in humans (in 1980 and 1981, by researchers in the United States and Japan, respectively) [8–10]. There are an estimated 10–20 million people infected with HTLV-1 worldwide, with endemic populations in Japan, Africa, the Caribbean Islands, central Australia, and South America [5, 11].

HTLV-1 induces ATL: mature malignant T-cell leukemias/lymphomas [5, 12–16]. ATL is known for having one of the worst prognoses of T-cell lymphomas [17, 18], although treatment options are slowly becoming available [19–24]. The oncogenesis of ATL is derived from the long-term survival of infected cells [15, 16]. Therefore, interrupting mechanisms for HTLV-1 persistence has the potential to provide a therapeutic strategy in the future [25].

### HTLV-1 proviral structure and oncogenesis

**Fig. 1:**
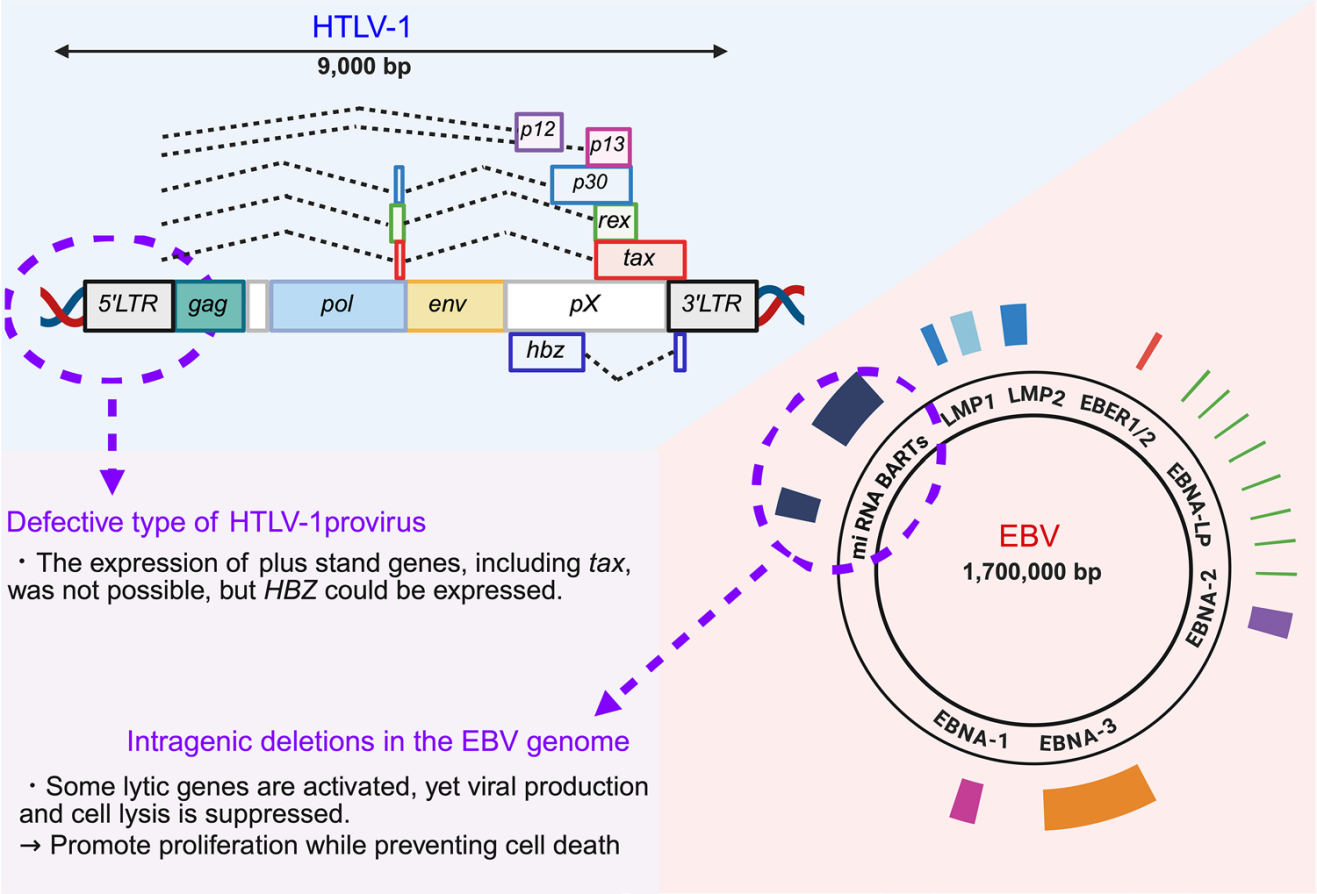
HTLV-1 proviral structure and EBV genome. Schemas showing the HTLV-1 provirus and the EBV genome. The HTLV-1 provirus encodes several regulatory (*tax* and *rex*) and accessory (*p12*, *p13*, *p30*, and *hbz)* genes in the *pX* region*.* In Type II latent EBV infection, the following are expressed: EBNA1, LMP1, LMP2, and the non-coding RNAs EBERs and BARTs. Both viruses commonly have deletions in their genomes, which result in a lack of gene expression. *HTLV-1* human T-cell leukemia virus type I, *LTR* long terminal repeat, *hbz* HTLV-1 bZIP factor, *EBV* Epstein-Barr virus, *LMP1* latent membrane protein 1, *LMP2* latent membrane protein 2, *EBER* Epstein-Barr Virus Early RNA, *EBNA* EBV nuclear antigen, *BARTs* *BamHI* A rightward transcripts

The HTLV-1 provirus is small in size (only approximately 9 kb), and encodes a long terminal repeat (LTR) at each end, structural genes (*gag*, *pol*, and *env*), several regulatory genes (*tax* and *rex*) and several accessory genes (*p12*, *p13*, *p30*, and *HTLV-1 bZIP factor [HBZ])* (Fig. 1) [5, 25]. The promoter region of plus-strand genes, including *tax*, is 5′LTR, whereas that of minus-strand gene, *HBZ*, is 3′LTR. Among these viral genes, *tax* and *HBZ* are well known as being responsible for oncogenesis [5, 14–16].

Tax controls several host signaling pathways, such as the nuclear factor-kappa B (NF-κB) pathway [26] and the Hippo signaling pathway [27]. Tax immortalizes human primary CD4+ T cells by activating the NF-κB pathway [28]. Further, Tax prevents their apoptosis [29, 30], and causes their proliferation [27]. Recently, Tax stability was found to depend on the activity of the tyrosine kinase KDR [31]. Tax contributes to both de novo and persistent infection.

On the other hand, HBZ plays a critical role mainly in persistent HTLV-1 infection. *HBZ* changes the phenotype of HTLV-1–infected cells to a regulatory T cell (Treg)-like phenotype by inducing the expression of Foxp3 [32–35], C–C chemokine receptor 4 (CCR4) [36], and T cell immunoreceptor with Ig and ITIM domains (TIGIT) [37]; these phenotypic changes help infected cells to evade the host immune system and facilitate their tissue migration.

Recently, key mechanisms by which HBZ contributes to persistence infection have become clearer [16, 25]. HTLV-1 hijacks a Treg associated signaling pathway to promote the proliferation of infected cells and evade host immunity as follows. HBZ enhances the production of IL-10, an immunosuppressive cytokine, to enable immune evasion [37]. Furthermore, IL-10 promotes the proliferation of HBZ-expressing cells [37–39]. Similarly, activation of the TGF-β signaling pathway by HBZ contributes not only to evading host immunity but also to enhancing clonal expansion of HTLV-1–infected cells [32, 40–42]. Interruption of these pathways might be a reasonable therapeutic strategy for ATL and HTLV-1 infection.

HBZ also induces the expression of other host genes associated with cell proliferation or apoptosis, including *CCR4* [36, 43], *BATF3* [44], *Bim* and *FasL* [45], *survivin* [46], *TAp73* [47], and *AK4/RHOC* [48].

Tax is highly immunogenic and easily recognized by cytotoxic T-lymphocytes (CTLs). Therefore, HTLV-1 establishes persistent infection partly by suppressing Tax expression. The fact that Tax expression occurs periodically but only transiently allows evasion infected cells from host immune attack and protects infected cells from apoptosis [30]. Interestingly, in half of ATL cases, Tax expression is impossible due to 5′LTR or internal deletions [49–51], methylation [52, 53], or nonsense mutations [40, 54]. In contrast to Tax, HBZ is less immunogenic, possesses various mechanisms for sustained expression, and is the most important contributor to persistent HTLV-1 infection [55–60].

In sum, viral genes, especially *HBZ*, are crucial for persistent infection because they promote the proliferation of infected cells and facilitate their evasion from the host immune system.

### Multi-step genomic/epigenetic alterations for development of ATL

Only about 5% of HTLV-1 carriers develop ATL during their lifetime, suggesting that persistent HTLV-1 infection alone is not enough to cause ATL. HTLV-1 replicates by inducing the proliferation of infected host T cells, and most infected clones are detectable for several years [61, 62]. During this long life, HTLV-1–infected cells experience a multi-step accumulation of somatic mutations and aberrant epigenetic changes, and then develop into ATL through ongoing clonal selection.

Kataoka et al. provided a detailed profile of the driver mutations in ATL [63, 64]. Multiple oncogenic driver mutations were identified in each patient with ATL, with a median of nine mutations per case [64]. Interestingly, somatic mutations in ATL were concentrated in the target genes of *HBZ* [65], including *CCR4*/*GATA3* [36, 43], *IKZF2* [35], *STAT3* [39], *TP53* [66, 67], *TP73* [47, 66], and *p300* [68]. Consistent with HTLV-1’s strategy for persistent infection, abnormalities contributing to host immune evasion were also identified: (1) structural variations disrupting the 3′ untranslated region of the *PD-L1* gene that upregulate *PD-L1* expression [69], (2) loss-of-function alterations preferentially affecting the *CIC* long isoform and thus selectively increasing the number of cells with the Treg phenotype (CD4+CD25+ Foxp3+) in the mouse model [64]. These mutations could help ATL cells and infected cells to survive for a long time.

Importantly, these driver mutations occur early, even in the HTLV-1 carrier state [61, 70–72]. One study showed that HTLV-1 carriers who later developed ATL already possessed premalignant clones with driver mutations 10 years prior to ATL onset, whereas age- and proviral load (PVL)-matched HTLV-1 carriers who did not develop ATL did not possess these driver mutations during the 10-year clinical observation period [61]. In another study, several carriers at high risk for ATL harbored driver mutations of ATL, such as *TP73, NOTCH1, CCR4, GPR183*, and *ARID2* [72].

Yamagishi et al. made a revealing study of multi-step ATL oncogenesis [70]. Through combined analysis of deep DNA sequencing and single-cell RNA sequencing, the clonal history of ATL was evaluated. A multi-step accumulation of mutations in the TCR, STAT3, and NOTCH signaling pathways was found to be important for establishing clone-specific transcriptomic abnormalities in the HTLV-1 carrier state and for accelerating the proliferative potential to generate highly malignant clones. Although high PVL is associated with the development of ATL [73, 74], most carriers with high PVL without driver mutations did not develop ATL at a median follow-up of 10 years [61], suggesting that the stepwise accumulation of mutations in these genes is key to the oncogenesis of ATL.

HBZ-transgenic mice have been identified as an ATL mouse model that develops T-cell lymphoma and systemic inflammation with a long latent period [33]. Since approximately 90% of ATL patients harbor somatic mutations associated with the TCR-NF-κB signaling pathway [63], the double transgenic mouse model combining HBZ and the CARD11 (E626K) mutation was investigated [75]. These double transgenic mice developed lymphoma earlier than HBZ-transgenic mice and exhibited an ATL-like phenotype, suggesting the fundamental roles of HBZ and the mutations in the TCR-NF-κB signaling pathway in ATL development [75].

Furthermore, epigenetic modifications, such as DNA methylation, histone modifications, and chromatin structural changes, also occur in the process of clonal selection and expansion that leads from HTLV-1 infection to ATL [14, 76–81]. In particular, Polycomb repressive complex 2 (PRC2)-dependent H3K27 trimethylation (H3K27me3) is reprogrammed across numerous gene promoters and gene regions throughout the genome in ATL [76–79].

### Summary of oncogenesis by HTLV-1

**Fig. 2:**
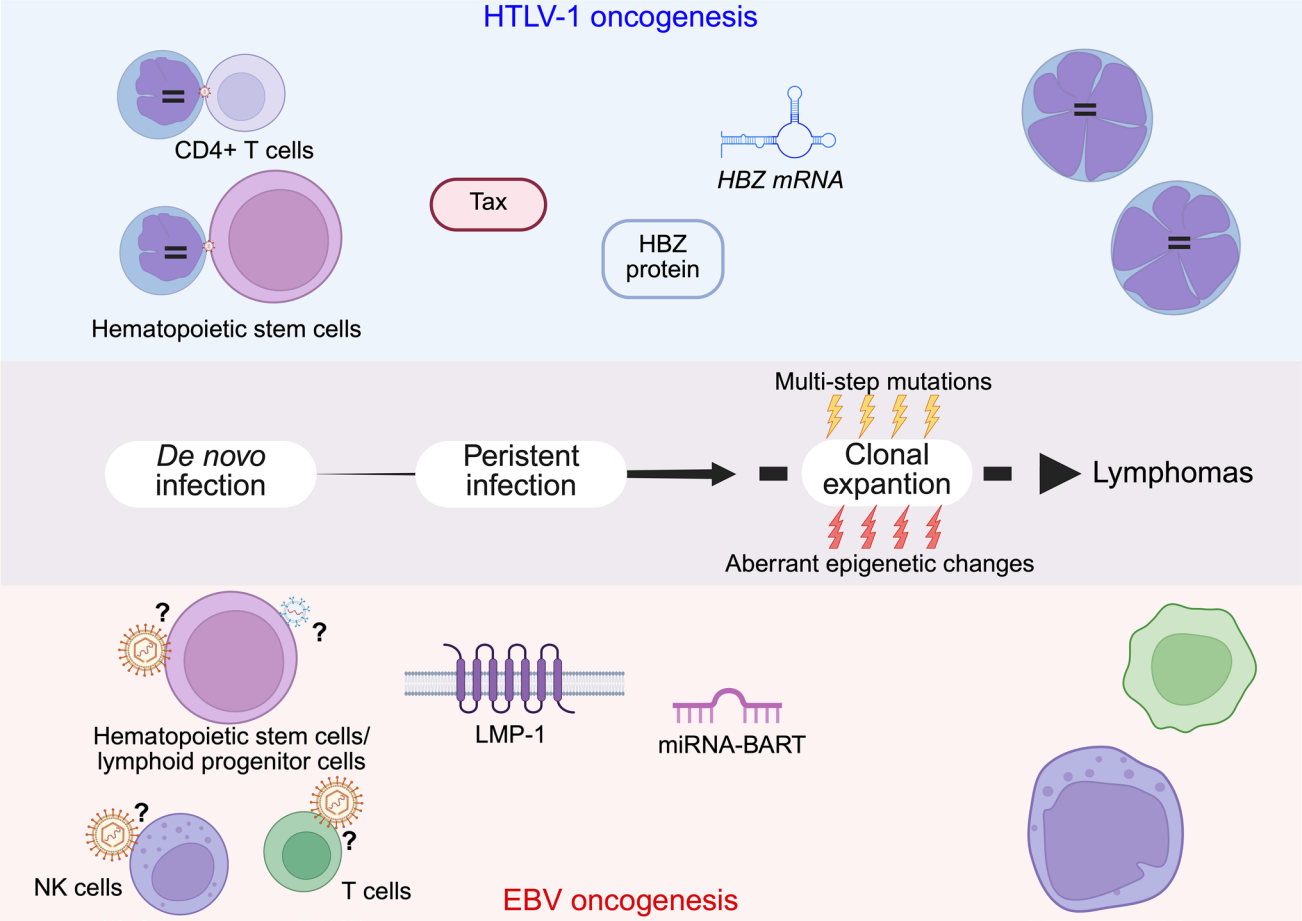
HTLV-1- and EBV-driven oncogenesis. (Top) HTLV-1 infects hematopoietic stem cells and/or CD4+ T cells, and the phenotype of clonally expanded HTLV-1–infected cells changes to Treg-like due to the action of HBZ. Tax and HBZ are the main contributors to de-novo and persistent infection, respectively. HTLV-1 maintains persistent infection by promoting the growth of infected cells and evading the host immune system. These actions lead to clonal expansion and subsequent lymphoma by the multistep accumulation of driver mutations and aberrant epigenetic changes. (Bottom) EBV is assumed to infect hematopoietic stem cells/lymphoid progenitor cells and/or T/NK cells. EBV latent genes, especially *LMP1*, modulate host signaling pathways to promote the survival of infected clones. Recurrent somatic mutations, particularly in *DDX3X*, and other epigenetic regulators drive immune evasion, malignant transformation and clonal evolution. *HTLV-1* human T-cell leukemia virus type I, *HBZ* HTLV-1 bZIP factor, *NK* natural killer cell, *Treg* regulatory T cell, *LMP1* latent membrane protein 1, *BARTs* *BamHI* A rightward transcripts

HTLV-1’s strategy for persistent infection is closely related to ATL oncogenesis (Fig. 2). Dysregulation of the molecules and the signaling pathways that are critical for survival and proliferation of infected cells is important for propagation of this virus in vivo. The prolonged survival of infected cells is linked to the accumulation of somatic mutations [63, 64] and aberrant epigenetic changes [76–81], and ongoing clonal selection based on capacity for growth promotion and immune evasion leads to ATL (Fig. 2).

The approved drugs for ATL target key oncologic molecules, such as CCR4 (mogamulizumab); IKZF1/3, IRF4 and the NF-κB pathway (lenalidomide); or epigenetic alterations (tucidinostat, valemetosetat). The anti-GARP monoclonal antibody, which depletes ATL cells by suppressing the activation of the TGF-β signaling pathway, is another potential novel therapeutic agent for ATL [82]. Further study of the underlying molecular mechanisms of ATL oncogenesis is crucial for the identification of additional therapeutic targets for ATL.

## Epstein-Barr virus induces extranodal NK/T-cell lymphoma and chronic active Epstein-Barr virus disease

EBV, a γ-herpesvirus, was the first oncovirus to be discovered in humans in 1964 [83]. EBV induces approximately 2% of all cancers, including lymphomas, epithelial cell cancers and gastric cancer [84].

Most adults are already infected with EBV, which usually remains asymptomatic, but it causes T/NK cell lymphomas in a very small number of people. ENKTCL is a mature T/NK-cell lymphoma with a poor prognosis that is particularly prevalent in East Asia and South America. Although recent advances in treatment have led to improved prognoses [85, 86], further therapeutic development is still required.

CAEBV is a mature T/NK-cell neoplasm characterized by persistent or recurrent systemic inflammation and clonal proliferation of EBV–infected T or NK cells [87]. The development of NK/T-cell lymphomas is more likely in patients with CAEBV [87]. Since chemotherapy cannot achieve long-term disease control, hematopoietic stem cell transplantation (HSCT) is considered the only curative treatment for CAEBV [88, 89].

To overcome these EBV-driven diseases with poor prognoses, it is important to understand how EBV causes them.

### EBV genes that promote oncogenesis

The EBV life cycle consists of two states: the lytic cycle, in which EBV reactivates due to immune suppression, destroys host cells, and produces new viral particles; and the latent cycle, in which the virus replicates its genome while remaining within the host cell [84]. Latent infection is subclassified into three distinct types—I, II, and III—based on the types of viral genes expressed. Type II latent infection, which is associated with the development of T-cell and NK-cell lymphomas, involves the expression of EBV nuclear antigen 1 (EBNA1), latent membrane protein 1 (LMP1), LMP2, and the non-coding RNAs Epstein-Barr Virus Early RNA (EBERs) and BARTs (Fig. 1) [84]. These EBV genes, especially *LMP-1*, act similarly to *tax* and *HBZ* in HTLV-1 in that they disrupt host signaling pathways, promote the survival of infected cells in the long term, and allow the virus to evade the host’s immune system.

The major EBV oncoprotein, LMP1, is in essence a constitutively active member of the tumor necrosis factor receptor (TNFR) superfamily. Its two cytoplasmic C-terminal activating regions, CTAR1 and CTAR2, recruit TNFR-associated signaling adaptors, such as TRAFs and TRADD. This interaction activates multiple downstream pathways, including the NF-κB [90–92], PI3K/Akt/mTOR [93–95], and JAK/STAT pathways [96–99], in a ligand-independent manner.

EBV thus induces the NF-κB-mediated survival signals in T and NK cells, contributing to lymphomagenesis [100]. Activation of the NF-κB and PI3K/Akt/mTOR pathways by LMP1 induces survivin, resulting in inhibition of apoptosis [101]. Furthermore, LMP1-mediated activation of the JAK/STAT pathway, particularly STAT3, induces the formation of NF-κB pathway subunits (the p50/Bcl-3 complex) and promotes cell proliferation [102]. LMP-1 also contributes to the survival and proliferation of T/NK cells by increasing CD137 expression [103].

In addition, LMP-1 contributes to host immune evasion [104]. Its activation of the JAK/STAT and AP-1 signaling pathways increases the expression of PD-L1, which is associated with T cell exhaustion [105]. In fact, PD-L1 overexpression is observed in the majority of ENKTCL patients (39%–100%), and high serum PD-L1 levels are associated with a poor prognosis in these patients [106, 107].

The *BamHI* A rightward transcript (BART) microRNA (miRNA-BART) is also associated with EBV-driven cancers and is involved in chronic inflammation and immune evasion. While miRNA-BARTs have been reported to exhibit few direct oncogenic mechanisms in T/NK cells themselves, it has been found that HL and DLBCL cells release miRNA-BARTs into the extracellular space via small extracellular vesicles, and these miRNA-BARTs act in *trans*, inducing a regulatory phenotype in macrophages and thus influencing the tumor microenvironment to allow evasion of immune surveillance [108].

Comprehensive EBV genome analysis identified intragenic deletions in the EBV genome that were commonly observed in CAEBV (35%), ENKTCL (43%), and EBV + DLBCL (71%), but not in infectious mononucleosis or posttransplant lymphoproliferative disorder, suggesting that common mechanisms are involved in the development of these hematological neoplasms [109]. Interestingly, these deletions often involve genes that are essential for miRNA-BART clusters or viral particle formation (e.g., BALF5). When these deletions occur, some lytic genes are activated, yet viral production and cell lysis is suppressed. This suggests that these deletions may promote proliferation while preventing cell death. Similar results were demonstrated by another group (in France) [110].

A UK cohort study reported that EBV deletion signatures detected in the blood were absent in saliva. The same deletions were detected at multiple time points during diagnosis but disappeared after successful treatment. These findings suggest that EBV deletion sequences may serve as useful biomarkers for disease clones [111].

On the other hand, another recent study demonstrated high expression levels of miRNA-BARTs both in cell lines and in EBV–infected T- or NK-cells from CAEBV patients, suggesting an oncogenic role of miRNA-BARTs [112]. Further studies are needed to conclusively determine the role of miRNA-BARTs in T/NK-cell lymphomagenesis.

### Driver mutations for development of EBV-associated T/NK cell lymphomas

Several recent studies have helped to elucidate the driver mutations of somatic genes that lead to ENKTCL and CAEBV. In one study of patients with ENKTCL, whole-exome sequencing (*n* = 25) and targeted sequencing (*n* = 80) identified the *DDX3X* gene as the most frequently mutated gene, followed by *TP53*, *MGA*, *STAT3*, *STAT5B, MLL2, ARID1A, EP300* and *ASXL3* [113]. Functionally, *DDX3X* mutations impair RNA-unwinding activity, enhance cell-cycle progression, and activate the NF-κB and MAPK pathways [113].

A recent study using 178 patients with ENKTCL identified that common gene alterations were *CD274, TP53, CDKN2A, ARID1A, HLA-A, MSN, BCOR,* and *STAT3* in that order. Furthermore, X-linked driver gene alterations (*MSN, BCOR, DDX3X*, and *KDM6A*) were frequently observed in cases harboring chromosome X losses. Mutation of the *MSN* gene promoted cell proliferation and activated the NF-κB pathway [114].

Interestingly, an ENKTCL mouse model was developed by NK-cell-specific *Trp53* deletion and expression of *LMP1* [115], suggesting that the development of ENKTCL is driven by a combination of viral gene activity and somatic mutations. This model was used to characterize the LMP1-induced pro-tumorigenic myeloid microenvironment, which was shown to be supported by CXC chemokine ligand 16 (CXCL16)-CXC chemokine receptor 6 (CXCR6) signaling. Furthermore, potential therapeutic targets, such as CXCL16, KLRG1, and MYC were identified.

Kataoka K, et al. reported that genetic aberrations of PD-L1/PD-L2 that caused a truncation of the 3′-untranslated region were frequently observed in ENKTCL (as they previously showed for ATL, as discussed above), suggesting that evasion from cellular immunity is critical for both diseases [116].

A multi-omics analysis of ENKTCL identified three robust, clinically relevant molecular subtypes: TSIM, MB, and HEA [117]. Each subtype is defined by a unique genomic, transcriptomic, and viral expression profile. For example, the TSIM subtype shows a higher expression level of the lytic gene *BALF3*; the HEA subtype shows a higher expression level of the lytic gene *BNRF1*; and the MB subtype displays lower levels of the latent gene *LMP1*. These findings are critical because they illuminate distinct pathogenic pathways and suggest subtype-specific therapeutic vulnerabilities. This information could guide the development of future mechanism-based treatments, such as PD-1 blockade for the TSIM subtype, MYC inhibitors for the MB subtype, and HDAC inhibitors for the HEA subtype.

In addition to these studies of ENKTCL driver mutations, several interesting studies have been made of potential driver mutations of CAEBV. A landmark study by Okuno et al. of EBV–infected cells from 80 patients with CAEBV demonstrated that the somatic gene most frequently mutated was the *DDX3X* gene (*n* = 14) [109]. This observation strongly suggests that CAEBV and ENKTCL share some common driver mutations. Furthermore, a multi-step accumulation of somatic mutations was observed in patients with CAEBV who eventually developed lymphoma. In the blastic crisis phase, *DDX3X* mutations were specifically detected in EBV–infected cells, implicating *DDX3X* in the progression from CAEBV to lymphoma. Notably, CAEBV patients harboring *DDX3X* mutations showed significantly poorer prognosis, indicating that this molecular subtype represents a more aggressive, lymphoma-like phenotype.

Another group (in China) also characterized a large cohort of predominantly adult-onset EBV-associated hemophagocytic lymphohistiocytosis (*n* = 46) and CAEBV (*n* = 73) [118]. This study found that genetic defects, both germline and somatic, were prevalent and associated with an unfavorable prognosis. Notably, aberrations in the RIG-I-like receptor (RLR) pathway (IFIH1 and/or DDX3X) were identified as key drivers linked to a higher EBV load and the upregulation of EBV-encoded oncogenes.

These studies identified the association of certain somatic mutations with CAEBV oncogenesis, especially for the lymphoma-like phenotype. However, since the majority of CAEBV cases in both studies lacked identifiable driver mutations, the disease likely represents a heterogeneous group with distinct molecular pathways of evolution. Further investigation is needed to clarify CAEBV oncogenesis.

Furthermore, recent studies revealed the epigenetic alterations both in ENKTCL and NK cell-type CAEBV [119, 120]. Significant DNA hypermethylation was identified in both diseases, resulting in robust epigenetic silencing of critical genes required for NK-cell differentiation, tumor suppression, and antiviral function [119, 120]. This profound epigenetic dysregulation creates a vulnerability that can be exploited for therapeutic purposes. Treatment with the DNA hypomethylating agent 5-azacytidine (5-aza) has been shown to disrupt the blockade, promote cellular differentiation, and improve survival in patient-derived xenograft models of both ENKTCL and high-risk CAEBV [119, 120].

### Summary of EBV oncogenesis

EBV-driven T/NK cell neoplasms, such as ENKTCL and the lymphoma-like phenotype of CAEBV, potentially share overlapping molecular and viral oncogenic mechanisms (Fig. 2). EBV latent genes, such as *LMP1*, together with certain deletions in the viral genome, result in continuous survival and proliferative signals while enabling infected clones to evade the immune system. Concurrently, recurrent somatic mutations, particularly in *DDX3X*, and other epigenetic regulators, cooperate with viral oncogenes to drive malignant transformation and clonal evolution. These findings suggest that EBV-associated T/NK cell neoplasms develop from the interplay of viral persistence, host genetic alterations, and the immune microenvironment (Fig. 2). Future studies integrating viral genomics, host epigenetics, and immunobiology are essential for fully elucidating EBV-driven oncogenesis and developing novel, mechanism-based therapies that target both the viral and cellular components of these aggressive disorders [121].

Recently, the targeting of EBV-associated oncogenic mechanisms, including PD-L1 for ENKTCL [122, 123] and the NF-κB pathway (bortezomib) [124] and the JAK/STAT pathway (ruxolitinib) [125] for CAEBV have been shown in clinical trials to be promising novel therapeutic strategies.

## Conclusions

HTLV-1 and EBV differ in terms of viral type, size, and the roles of their viral genes, but both cause lymphomas with poor prognoses. A common oncogenic mechanism involves a complex interplay of host immune evasion and disruption of host signaling pathways—strategies for persistent viral infection—alongside the accumulation of somatic mutations and epigenetic abnormalities within infected cells (Fig. 2). A detailed elucidation of these mechanisms is expected to lead to the development of novel therapies for these NK/T-cell lymphomas.

## Data Availability

The manuscript has no associated data. Therefore, no data relating to this manuscript is available.
